# Effect of β-Aminobutyric Acid on Disease Resistance Against *Rhizopus* Rot in Harvested Peaches

**DOI:** 10.3389/fmicb.2018.01505

**Published:** 2018-07-10

**Authors:** Jing Wang, Shifeng Cao, Lei Wang, Xiaoli Wang, Peng Jin, Yonghua Zheng

**Affiliations:** ^1^College of Food Science and Technology, Nanjing Agricultural University, Nanjing, China; ^2^College of Biological and Environmental Sciences, Zhejiang Wanli University, Ningbo, China; ^3^College of Agriculture, Liaocheng University, Liaocheng, China; ^4^School of Life Science and Food Engineering, Huaiyin Institute of Technology, Huai’an, China

**Keywords:** *Prunus persica* fruit, β-aminobutyric acid, *Rhizopus stolonifer*, induced resistance, energy status

## Abstract

The effect of β-aminobutyric acid (BABA) on *Rhizopus* rot produced by *Rhizopus stolonifer* in harvested peaches and the possible action modes were investigated. Treatment with 50 mmol L^−1^ of BABA resulted in significantly lower lesion diameter and disease incidence compared with the control. The activities of defense-related enzymes chitinase and β-1,3-glucanase were notably enhanced by this treatment. Meanwhile, BABA treatment also increased lignin accumulation and maintained higher energy status in peaches by enhancing activities of enzymes in the phenylpropanoid and energy metabolism pathways. Semiquantitative reverse transcription PCR results indicated that the transcription of four defense-related genes was substantially and rapidly enhanced only in that BABA-treated fruit upon inoculation with the pathogen. Thus, our results demonstrated that BABA was effective on controlling *Rhizopus* rot by inducing disease resistance, which includes the increase in gene transcription and activity of defense-related enzymes, the enhancement of cell wall strength, and the maintenance of high energy status in *Prunus persica* fruit. Moreover, the disease resistance induced by BABA was demonstrated through priming model rather than direct induction.

## Introduction

Peaches [*Prunus persica* (L.) Batsch] suffer a short shelf life at room temperature after harvest, due to their rapid ripening and high susceptibility to pathogens, including *Rhizopus stolonifer* Ehrenb.: Fr., *Monilinia* spp., *Botrytis cinerea* Pers.: Er., and *Penicillium expansum* Link (Usall et al., 2015). Among these diseases, it is reported that *Rhizopus* rot caused by *R. stolonifer* is the most destructive disease in post-harvest stone fruit including peaches in China (Fan and Tian, 2000). In order to enhance disease resistance and extend shelf life of peaches, a number of physical or chemical treatments such as methyl jasmonate (MeJA; Jin et al., 2009), heat (Liu et al., 2012), high oxygen (Wang et al., 2005), and benzo-(1,2,3)-thiadiazole-7-carbothioic acid *S*-methyl ester (BTH; Liu et al., 2005; Cao et al., 2011) have been explored.

In general, energy plays an important role in maintaining membrane integrity, which is essential to plant cells. When plants are suffered from extreme or sustained energy deficiency, membrane damage cannot be repaired, and cells, tissues, or entire plants subsequently will die (De Block et al., 2005). It is well known that biotic and abiotic stresses result in great energy depletion, which is associated with the reduction of disease resistance (Jiang et al., 2007; Yi et al., 2010; Chen et al., 2014). Therefore, maintaining a high-energy status is essential in disease resistance. It has been reported that the application of exogenous adenosine triphosphate (ATP) improved the energy status of litchi fruit and suppressed disease development caused by *Peronophythora litchii* (Yi et al., 2008). Cao et al. (2014) also found that the maintenance of ATP content was an important mechanism by which MeJA treatment induced disease resistance in post-harvest loquat fruit.

Defense priming in plants was first noted in 1933 and was initially termed as “sensitization” (Chester, 1933). Recently, priming is considered as a common phenomenon that plants do not exhibit any detectable defense responses after treatment with a priming inducing agent; however, a faster and stronger activation of defense responses is initiated only after they have been subjected to a subsequent stress (Conrath et al., 2002, 2006; Conrath, 2011). Recent studies showed that elicitors such as *Bacillus cereus* AR156 (Wang et al., 2013b; Wang X.L. et al., 2014) and MeJA (Wang et al., 2015; Saavedra et al., 2017) primed disease resistance in post-harvest fruits, thereby resulting in faster and stronger defense responses against pathogens. The small molecule β-aminobutyric acid (BABA), which is considered as a potential chemical inducer of disease resistance, has been investigated for many years (Thevenet et al., 2017). Previous reports demonstrated that the application of BABA treatment induced local or systemic resistance against various plant pathogens (Justyna and Ewa, 2013; Thevenet et al., 2017). Moreover, it has been shown that BABA can induce disease resistance and suppress disease incidence in a number of post-harvest fruits. For instance, BABA treatment induced disease resistance and reduced blue mold rot caused by *P. expansum* in grapefruit (Porat et al., 2003) and apples (Quaglia et al., 2011; Zhang et al., 2011), and the *anthracnose* rot caused by *Colletotrichum gloeosporioides* in mangoes (Zhang et al., 2013). However, no study has evaluated the efficacy of BABA against *Rhizopus* rot in peaches. In addition, it is unclear whether priming is a common phenomenon in BABA-induced resistance. Thus, our aims were to assess the effect of BABA on controlling *Rhizopus* rot caused by *R. stolonifer* in peaches after harvest and to investigate possible mechanistic models involved in disease resistance.

## Materials and Methods

### Pathogen

*Rhizopus stolonifer* was purified from infected peaches and cultured at 26°C on potato dextrose agar (PDA) medium for 2 weeks. The petri dishes were flushed with sterile distilled water with Tween 80 (0.05%) to collect *R. stolonifer* spores, and adjust the suspension to 1 × 10^5^ spores per milliliter with water described above. The spore suspension was maintained at 4°C for no more than 2 h prior to use.

### Plant Material and Treatments

Peaches [*P. persica* (L.) Batsch cv. Baifeng] were picked in a commercial garden (latitude 32°02′N; longitude 118°51′E) in Nanjing, Jiangsu province, at the firm-mature stage (Fernández-Trujilio et al., 1998) and transported to the laboratory within 2 h. In the laboratory, fruit free of wounds and rot were selected for homogeneous size, color, and maturity stage and divided randomly into four groups for four treatments: Mock, BABA, Inoculation, and BABA + Inoculation. The fruit was sterilized with 70% ethanol around the fruit equator and air-dried for 1 h prior to wounding.

Each peach was punched on two sides around the equatorial section with a sterilized nail to create two uniform wounds (2 mm wide and 4 mm deep). For the Mock and Inoculation groups, 30 μL of sterile distilled water was injected into each hole. For the BABA and BABA + Inoculation groups, the fruit were injected with 30 μL of 50 mmol L^−1^ BABA (Sigma, St. Louis, MO, United States). This specific concentration was chosen according to our preliminary experiment, which indicated that 50 mmol L^−1^ BABA was the most effective concentration comparing to the ones at 5 and 100 mmol L^−1^. The fruit were air-dried and placed in 330 mm × 220 mm × 60 mm plastic containers at 20°C. Six hours later, the Inoculation and BABA + Inoculation groups were challenge-inoculated with 15 μL of a *R. stolonifer* spore suspension (1 × 10^5^ spores per mL) in each wound. All peaches then were stored at 20°C for 60 h to allow for disease development. Three replicates of 48 fruit each were used per treatment, and eight fruit from each replicate were used at each time point for different analyses.

To investigate the efficacy of BABA on controlling *Rhizopus* rot caused by *R. stolonifer* infection in harvested peaches and its relation to disease resistance induction by BABA, disease incidence, and lesion diameter on each fruit wound were observed at 12, 24, 36, 48, and 60 h post inoculation in the Inoculation and BABA + Inoculation groups. Meanwhile, fruit flesh tissue from these two groups was collected within 10 mm around decay area by freezing in liquid nitrogen and storing at −20°C for lignin content, energy status, and enzyme assays.

For further revealing whether the BABA induced disease resistance against *Rhizopus* rot is associated with priming of defense responses in peaches, fruit samples from all the four groups were collected at 3, 6, 12, and 24 h post inoculation within 10 mm around decay area in pathogen challenged fruit (Inoculation and BABA + Inoculation) or equal position of healthy area in pathogen-free fruit (Mock and BABA) at the equator of peach fruit. Semiquantitative reverse transcription PCR (RT-PCR) was used to analyze the expression patterns of the four defense-related genes β-1,3-glucanase (*GNS*), chitinase (*CHI*), non-expressor of pathogenesis-related protein1 (*NPR1-like*), and pathogenesis-related protein (*PR-like*).

### Evaluation of Decay

Eight fruit from each triplicate were used for decay evaluation at each time point. Fruit with a visibly diseased area more than 1 mm wide around the wound were considered decayed. Lesion diameter was measured using a vernier caliper. Disease incidence was determined according to the following formula:

$$\text{Disease incidence }(\%)=\frac{\text{decayed fruits}}{\text{total fruits}}\times 100\%$$

### Enzyme Assays

A crude enzyme extracted from 1 g of frozen flesh tissue with 50 mmol L^−1^ of sodium acetate buffer for detecting β-1,3-glucanase (GLU) and chitinase (CHI) activities was prepared. GLU and CHI activity was determined referred to the procedure of Abeles et al. (1971). One unit of GLU activity was expressed as the increase in absorbance of 0.001 at 540 nm. One unit of CHI activity was expressed by the production of 1 mg glucose per minute.

Phenylalanine ammonia lyase (PAL) activity was evaluated according to Cheng and Breen (1991) with some modification. One unit of PAL activity is defined as the quantity of enzyme that causes a 0.01 increase in absorbance at 290 nm in 1 h. 4-Coumaryl CoA ligase (4CL) activity was assayed as the protocol of Knobloch and Hahlbrock (1977). The activity of 4CL is determined as the quantity of enzyme that resulted in a 0.01 increase in absorbance per minute. Cinnamate 4-hydroxy (C4H) activity was evaluated as the protocol of Lamb and Rubery (1975). We measured 4-hydroxy-*trans*-cinnamic acid production by the absorbance at 340 nm compared to a reference extract containing *trans*-cinnamic acid that was measured using the same procedure.

Five grams of fruit flesh was homogenized with 10 mL Tris–HCl buffer (pH 7.5) and filtered with four-layer nylon gauze. The homogenate was centrifuged at 4,000 *g* for 10 min at 4°C, and the supernatant was centrifuged at 12,000 *g* for 10 min at 4°C. The ultimate supernatant was crude mitochondria enzyme extract that was used for measurement of activities of enzymes related to energy metabolism. ATPases activity was assayed by determining inorganic phosphorus product by the catalytic of ATP reaction to adenosine diphosphate (ADP) as the method of Jin et al. (2013). For H^+^-ATPase activity assay, 1.0 mL reaction system was 30 mmol L^−1^ Tris–HCl (pH 8.0) containing 3 mmol L^−1^ Mg_2_SO_4_, 0.1 mmol L^−1^ Na_3_VO_4_, 50 mmol L^−1^ NaNO_3_, 50 mmol L^−1^ KCl, and 0.1 mmol L^−1^ ammonium nitrate. Enzyme crude extract (0.05 mL) and 0.1 mL ATP–Tris was added into the mixture to start the reaction and incubated at 37°C water bath for 20 min. The reaction was terminated by 0.1 mL 55% TCA. Ca^2+^-ATPase activity assay method was similar to H^+^-ATPase. In this sense, 3 mmol L^−1^ Mg_2_SO_4_ was replaced by 3 mmol L^−1^ Ca(NO_3_)_2_. Ca^2+^-ATPase activity was expressed by dispersion activity with and without Ca(NO_3_)_2_. One unit of H^+^-ATPase and Ca^2+^-ATPase activities were expressed by the release of phosphorus per minute.

Cytochrome *c* oxidase (CCO) and succinate dehydrogenase (SDH) activity was measured referred to the procedure of Ackrell et al. (1984) with modifications. A total of 2.8 mL potassium phosphate buffer (0.2 mol L^−1^, pH 7.4) containing 0.2 mol L^−1^ sodium succinate and 0.9 mmol L^−1^ 2,6-dichlorophenolindophenol sodium salt was incubated at 30°C for 5 min. Enzyme extract (0.1 mL) and phenazine methosulfate (0.1 mL) were added to the reaction systems successively. Absorption was detected at 600 nm. One unit of SDH activity was defined as an increase of 0.01 per minute and expressed as U mg^−1^ protein. For CCO activity measurement, 0.2 mL enzyme extract was added with 0.02 mL 0.04% (w/v) cytochrome *c* and 2 mL ultrapure water. The whole system was bathed at 37°C water for 2 min. Then, 0.5 mL 0.4% (w/v) dimethyl-*p*-phenylenediamine was added to the mixture and the absorption was determined at 510 nm. One unit of CCO activity was defined as an increase of 0.01 per min and expressed as U mg^−1^ protein.

### Measurement of Lignin Content

Lignin content was quantified gravimetrically as the protocol of Femenia et al. (1998) with little modification. Ten grams of tissue were ground with 10 mL of distilled water and homogenized in 20 mL of concentrated sulfuric acid overnight. The mixture was then diluted to 250 mL and boiled for 2.5 h. The homogenate was filtered with hot water (90°C) until the effluent was not acidic. The remaining sediment was dried at 105°C to a constant mass. The mass was noted and expressed as a percentage.

### Determination of ATP, ADP, and AMP Contents and Energy Charge

Adenosine triphosphate, ADP, and adenosine monophosphate (AMP) were quantified using the protocol of Liu et al. (2006). Two grams of frozen flesh was homogenized with 6.0 mL perchloric acid and centrifuged at 12,000 *g* for 15 min. The supernatant was filtered by 0.45-μm filter membrane. A 20-μL sample was taken for HPLC analysis. A mixture of ATP, ADP, and AMP was injected onto the HPLC as an external standard solution under the same conditions. The energy charge was equivalent as the function according to Pradet and Raymond (1983).

$$\text{Energy charge}=\frac{\text{ATP}+\frac{1}{2}\text{ ADP}}{\text{ATP}+\text{AMP}+\text{ADP}}$$

### Determination of Defense-Related Gene Expression by Semiquantitative RT-PCR

Fruit flesh tissue was collected from peaches in Mock, BABA, Inoculation, and BABA + Inoculation groups at 3, 6, 12, and 24 h after inoculation. For each replicate at different sampling time, 4 g of frozen flesh tissue was powdered in liquid nitrogen to get total RNA according to the method of cetyltrimethyl ammonium bromide (Chang et al., 1993). Total RNA (100 ng) was reverse-transcribed with HisScript^®^ 1st Strand cDNA Synthesis Kit (Vazyme, Jiangsu, China). Short and conserved segments of *GNS* (Genebank: U49454.1), *CHI* (Genebank: AF206635.1), *NPR1-like* (Genebank: DQ149935.1), and *PR-like* (Genebank: AF362989.1, known as pathogen-related protein class 4) were cloned with 2 × Taq Master Mix kit (Vazyme, Jiangsu, China) using specific degenerate primers obtained from the SBS Genetech Co., Ltd. (Beijing, China). Semiquantitative RT-PCR was conducted as previously reported (Wang et al., 2013b). Independent 35 cycles were performed using 1 μl of cDNA samples to make sure linear amplification. The cycling conditions were conducted as the following program: 94°C – 5 min (1 cycle); 94°C – 30 s, 55°C – 30 s, and 72°C – 60 s/kb (35 cycles); 72°C – 7 min (1 cycle). *18S-rRNA* (Genebank: L28749.1) was set as the housekeeping gene for reference. Primers used in semiquantitative RT-PCR were shown in **Table 1**.

**Table 1:** Primers used in this assay for semiquantitative RT-PCR.

Genes	Primer sequence (5′→3′)	Product length (bp)
*GNS*	Forward: ATTTCTCTTGCTGGTCTTG	528
	Reverse: CTCTGGGGTCTTTCTATTCT	
*CHI*	Forward: GTGGAAAAGCAATAGGGGAG	244
	Reverse: TTCCAGCCCTTACCACAT	
*PR-like*	Forward: ATCAACTGGGACTTGCGTACT	317
	Reverse: TAGTCGCCACAGTCAACAAAG	
*NPR1-like*	Forward: GACCCAAACATGCCAGCAGTG	375
	Reverse: ATCCTTCGGCCTTGTCAACCT	
*18S-rRNA*	Forward: ATGGCCGTTCTTAGTTGGTG	356
	Reverse: GTACAAAGGGCAGGGACGTA	

### Statistical Analysis

All values were shown as the means ± standard error (SE) of triplicate assays. Two-way analysis of variance (ANOVA) was conducted with SPSS version 17.0 (SPSS Inc., Chicago, IL, United States) to evaluate the effects of treatment and storage time. Duncan’s multiple range tests were used to separate mean with *P* < 0.05 (regarded as significant).

## Results

### Effects of BABA on Controlling *Rhizopus* Rot in Peaches

**FIGURE 1:**
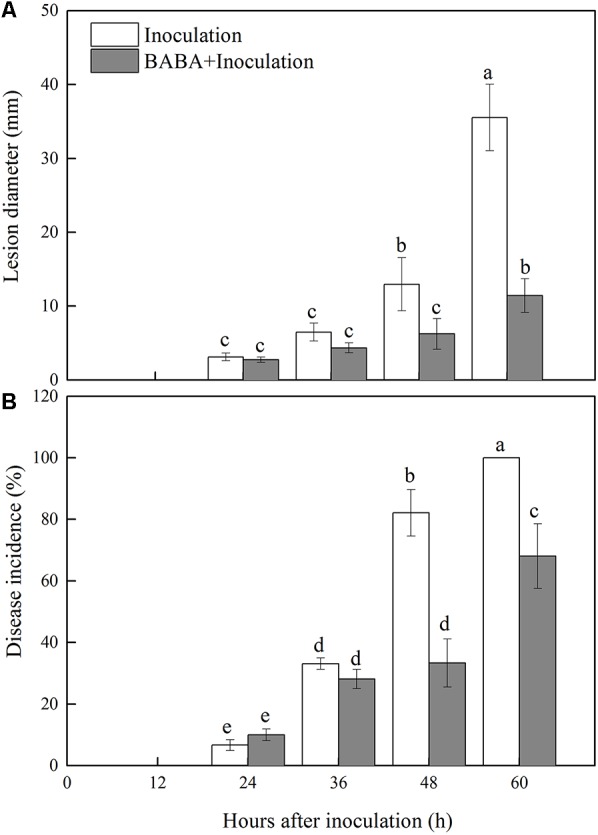
Changes in lesion diameter **(A)** and disease incidence **(B)** in peach fruit inoculated with *Rhizopus stolonifer* (Inoculation) or pretreated with 50 mM BABA and then inoculated with *R. stolonifer* (BABA + Inoculation) during storage at 20°C. Each column represents the mean of triplicate samples. Vertical bars represent the standard errors of the means. Letters without the same letter above the bars indicate significant differences at *P* < 0.05.

Decay symptoms resulting from *R. stolonifer* appeared in Inoculation and BABA + Inoculation peaches after 24 h post-inoculation. However, the lesion diameter and disease incidence of *Rhizopus* rot in BABA + Inoculation peaches were significantly (*P* < 0.05) lower than inoculation group from 36 to 60 h at 20°C (**Figure 1**). BABA treatment lowered lesion diameter and disease incidence by 67.88 and 31.94%, respectively, at 60 h post-inoculation compared with those in the Inoculation group (**Figure 1**).

### Effects of BABA Treatment on Chitinase and β-1,3-Glucanase Activities in Peaches

**FIGURE 2:**
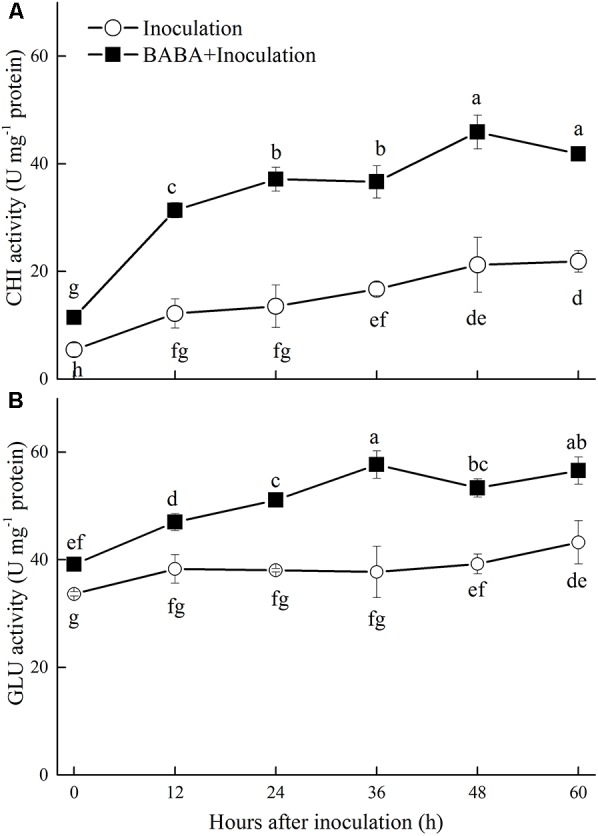
Changes in chitinase (CHI) **(A)** and β-1,3-glucanase (GLU) **(B)** activities in peach fruit inoculated with *Rhizopus stolonifer* (Inoculation) or pretreated with 50 mM BABA and then inoculated with *R. stolonifer* (BABA + Inoculation) during storage at 20°C. Data are expressed as the mean of triplicate samples. Vertical bars represent the standard errors of the means. Letters without the same letter above the bars indicate significant differences at *P* < 0.05.

Chitinase and GLU are important enzymes for the catalytic hydrolysis of fungal cell walls. As shown in **Figure 2**, the activities of both enzymes increased during storage. BABA treatment induced and maintained significantly (*P* < 0.05) higher activities of these two enzymes than the untreated fruit.

### Effects of BABA Treatment on Lignin Content and Related Enzymes in Post-harvest Peaches

**FIGURE 3:**
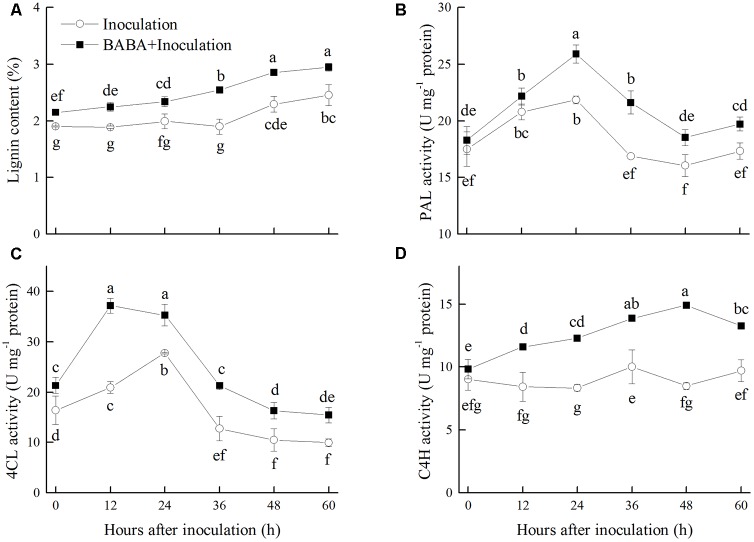
Changes in lignin content **(A)**, activities of phenylalanine ammonia lyase (PAL) **(B)**, 4-coumaryl CoA ligase (4CL) **(C)**, and cinnamate 4-hydroxy (C4H) **(D)** in peach fruit inoculated with *Rhizopus stolonifer* (Inoculation) or pretreated with 50 mM BABA and then inoculated with *R. stolonifer* (BABA + Inoculation) during storage at 20°C. Data are expressed as the mean of triplicate samples. Vertical bars represent the standard errors of the means. Letters without the same letter above the bars indicate significant differences at *P* < 0.05.

Lignin content in peaches accumulated gradually during storage at 20°C, which was significantly induced by BABA treatment. Lignin content in the BABA treatment group was 16.67% higher than that in the control fruit after 60 h of storage (**Figure 3A**). PAL, 4CL, and C4H, the key enzymes responsible for the first steps of lignin biosynthesis in the phenylpropanoid pathway, were induced by BABA treatment during the storage. The activities of PAL, 4CL, and C4H were 13.77, 55.31, and 36.50% higher than the control group, respectively, at the end of the storage (**Figures 3B–D**).

### Effects of BABA Treatment on Energy Status in Peaches

**FIGURE 4:**
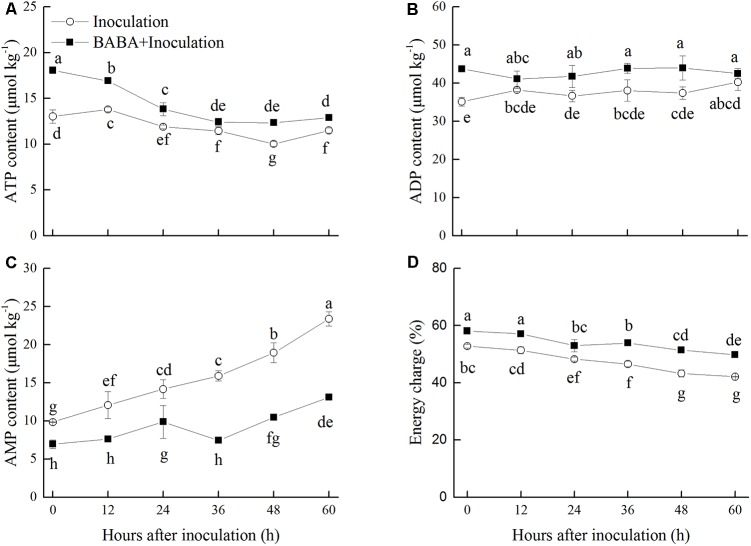
Changes in contents of adenosine triphosphate (ATP, **A**), adenosine diphosphate (ADP, **B**), adenosine monophosphate (AMP, **C**), and energy charge **(D)** in peach fruit inoculated with *Rhizopus stolonifer* (Inoculation) or pretreated with 50 mM BABA and then inoculated with *R. stolonifer* (BABA + Inoculation) during storage at 20°C. Data are expressed as the mean of triplicate samples. Vertical bars represent the standard errors of the means. Letters without the same letter above the bars indicate significant differences at *P* < 0.05.

Adenosine triphosphate content in peaches exhibited a gradually decreasing trend, whereas ADP content was maintained at a stable level in peaches inoculated with *R. stolonifer* (**Figures 4A,B**). Significantly (*P* < 0.05) higher levels of ATP were observed in BABA-treated peaches in comparison with the control group with the exception at 36 h. Meanwhile, ADP level showed the similar change as ATP level. ADP content accumulated at the initial storage time after BABA treatment and showed significantly (*P* < 0.05) higher levels at 36 and 48 h than that in control peaches. BABA treatment significantly (*P* < 0.05) limited the increase of AMP content during storage (**Figure 4C**). The energy charge in peaches declined over time during storage. However, the values of the energy charge in BABA-treated peaches were significantly (*P* < 0.05) higher compared to those in untreated fruit (**Figure 4D**).

### Effects of BABA Treatment on Ca^2+^-ATPase, H^+^-ATPase, SDH, and CCO Activities in Peaches

**FIGURE 5:**
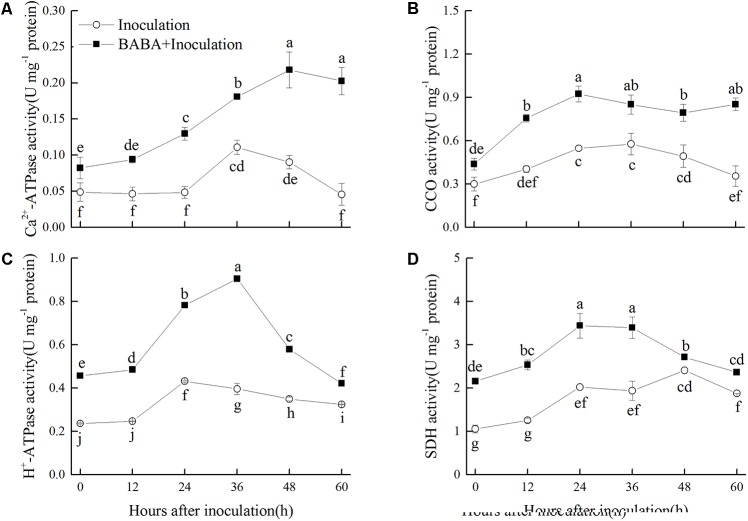
Changes in activities of Ca^2+^-ATPase **(A)**, cytochrome *c* oxidase (CCO) **(B)**, H^+^-ATPase **(C)**, and succinate dehydrogenase (SDH) **(D)** in peach fruit inoculated with *Rhizopus stolonifer* (Inoculation) or pretreated with 50 mM BABA and then inoculated with *R. stolonifer* (BABA + Inoculation) during storage at 20°C. Data are expressed as the mean of triplicate samples. Vertical bars represent the standard errors of the means. Letters without the same letter above the bars indicate significant differences at *P* < 0.05.

Activities of Ca^2+^-ATPase and H^+^-ATPase in peaches of Inoculation group increased slightly and peaked at 36 and 24 h, respectively, and then decreased during storage. BABA treatment stimulated the enhancement of Ca^2+^-ATPase and H^+^-ATPase activities, and kept them at significantly (*P* < 0.05) higher levels compared with the non-BABA-treated fruit over the entire storage period (**Figures 5A,C**). CCO activity increased at the first 24 h and then decreased gradually during the remaining storage time. The activity of CCO was significantly (*P* < 0.05) induced by BABA treatment within the whole storage (**Figure 5B**). As shown in **Figure 5D**, SDH activity increased during the first 48 and 24 h in the Inoculation and BABA + Inoculation groups, respectively, and decreased afterwards. Significantly higher SDH activity was observed in BABA-treated peaches (*P* < 0.05).

### Effects of BABA Treatment and *R. stolonifer* Inoculation on the Expression of Defense-Related Genes in Peach Fruit

**FIGURE 6:**
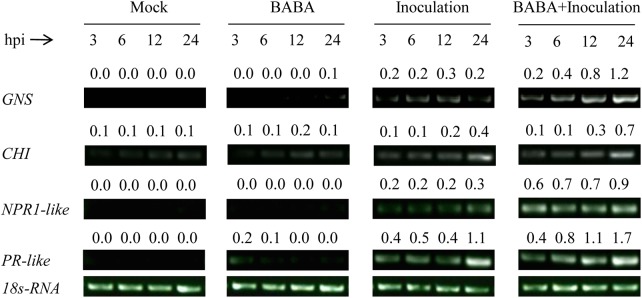
Changes in expression levels of the defense related genes β-1,3-glucanase (*GNS*), chitinase (*CHI*), non-expressor of pathogenesis-related protein1 (*NPR1-like*), and pathogenesis-related protein (*PR-like*) in peach fruit after inoculation with sterile distilled water (Mock), *Rhizopus stolonifer* (Inoculation), 50 mM BABA (BABA), or with 50 mM BABA and inoculated with *R. stolonifer* (BABA + Inoculation) during storage at 20°C. Semiquantitative reverse transcription-polymerase chain reaction (RT-PCR) was conducted using *18S-rRNA* as the internal control. hpi, hours post-inoculation.

The transcription of the four defense-related genes *GNS*, *CHI*, *NPR1-like*, and *PR-like* remained at a very low level in peach fruit only treated with BABA or sterile distilled water (Mock), while the transcription was slightly increased in fruit only inoculated with *R. stolonifer*. However, the transcription of the four genes in peaches both treated with BABA and inoculated with *R. stolonifer* was significantly enhanced and kept at higher level during storage compared with the other three treatments (**Figure 6**), which indicated BABA treatment induced higher expression of the four defense related genes in peaches upon inoculation with the pathogen of *R. stolonifer*.

## Discussion

Our study found that BABA treatment markedly reduced the development of *Rhizopus* rot in peaches during storage at 20°C, which suggested that disease resistance in peaches was enhanced by BABA. CHI and GLU are the crucial enzymes that degrade the cell walls of pathogens. Increased transcript accumulation of genes encoding these two enzymes and enhancement of enzyme activities have been extensively observed in the induction of disease resistance in post-harvest fruits (Cao et al., 2011; Liu et al., 2012; Wang et al., 2013a,b; Saavedra et al., 2017). Zhang et al. (2011) revealed that BABA treatment induced a remarkable enhancement in CHI and GLU activities in apples against blue mold decay. In the present study, BABA treatment significantly increased the gene transcription and activity of these two enzymes and inhibited *Rhizopus* rot in peaches, which suggested that the control of the disease by BABA was resulted from the induction of these two defense-related enzymes.

Lignin biosynthesis and lignification of cell wall play an important role in plant defense against pathogen invasion (Bhuiyan et al., 2009). PAL, C4H, and 4CL are three key enzymes responsible for the first steps of lignin biosynthesis in the phenylpropanoid pathway (Ferrer et al., 2008). The accumulation of gene transcripts for these three enzymes or the increase in their activities in response to elicitors has been observed in different harvested fruits. For example, Wang et al. (2015) reported the inhibition of decay development in sweet cherries by MeJA treatment was associated with the increased accumulation of *PAL* transcripts. Hershkovitz et al. (2012) found that the biocontrol agent *Metschnikowia fructicola* increased the abundance of plant defensive compounds via increasing expression of the genes encoding *PAL* and *4CL* in grapes. In addition, acibenzolar-*S*-methyl treatment induced the increase of PAL, C4H, and 4CL activities and thereby activated the phenylpropanoid pathway and prevented pathogenic invasion in muskmelon (Liu et al., 2014). In our present study, BABA treatment significantly increased PAL, C4H, and 4CL activities and consequently promoted the accumulation of lignin, which could contribute to the delay of *Rhizopus* rot development.

Energy status is a fundamental feature of ripening and senescence in harvested horticultural crops (Jiang et al., 2007). As a non-specific response in the host, the enhancement of ATP content plays a vital role in disease defense (Yi et al., 2010). Along with less disease development, higher energy status in post-harvest fruit will contribute to the production of natural compounds related to defense such as phytoalexins, and the enhancement of *PR-like* activity (Yi et al., 2010). Thus, the exogenous application of inducers that improve energy status may be an effective way to inhibit post-harvest diseases. Yi et al. (2010) reported that exogenous ATP treatment improved the energy status of harvested litchi fruit and inhibited disease development caused by *P. litchii*. The disease resistance in loquat fruit induced by MeJA was also found to be related to higher ATP content (Cao et al., 2014). Therefore, in our present study, the maintenance of high ATP level and energy charge with BABA treatment was crucial to disease resistance induction in peaches inoculated with *R. stolonifer*.

Adenosine triphosphate, ADP, and AMP contents are relevant to the enzymes activities in energetic metabolism pathways, involving Ca^2+^-ATPase, H^+^-ATPase, SDH, and CCO. Ca^2+^-ATPase is responsible for maintaining low cytoplasmic Ca^2+^, which is necessary for cellular balance (Palmgren and Harper, 1999). H^+^-ATPase produces a chemiosmotic H^+^-gradient and establishes a pH gradient around the plant plasma membrane, playing an important role in energy metabolism (Palmgren and Harper, 1999). SDH generates ATP by catalyzing succinate oxidized to fumarate, while CCO is the ultimate decisive enzyme in the respiratory electron transport system (Millar et al., 1995). All of these enzymes are essential for energy supply and maintenance of normal mitochondrial function. It has been demonstrated that alleviation of chilling injury in post-harvest fruit is relevant to increase of energy metabolism enzymes activities. Jin et al. (2013, 2014) discovered that MeJA or oxalic acid reduced chilling injury of peaches during cold storage by enhancing the activities of ATPases, SDH, and CCO. In our present study, we showed that BABA treatment maintained higher activities of Ca^2+^-ATPase, H^+^-ATPase, SDH, and CCO and thereby a higher energy status was observed in treated peaches compared to control fruit which plays a crucial role in inducing disease resistance.

Plant immunity consists of induced systemic resistance (ISR) and systemic acquired resistance (SAR). For a long time, it has been assumed that protection by induced disease resistance is based on direct activation of defense responses. Recently, priming is considered as a mechanism that is common to different types of induced disease resistance in plants, based on studies on field crops and model plants (Conrath, 2011). More recent studies have demonstrated that priming might also be a common phenomenon of induced disease resistance in post-harvest fruits. For examples, *B. cereus* AR156 induced disease resistance against *Rhizopus* rot in peach fruit and anthracnose rot in loquat fruit by priming of defense responses (Wang et al., 2013b; Wang X.L. et al., 2014). MeJA primed disease resistance against *Penicillium citrinum* in Chinese bayberries (Wang K.T. et al., 2014), *P. expansum* in sweet cherry fruit (Wang et al., 2015), and *B. cinerea* in table grapes and strawberries (Jiang et al., 2015; Saavedra et al., 2017). Yu et al. (2014) found that γ-aminobutyric acid induced disease resistance against *P. expansum* in pear fruit through priming of defense responses. In line with these results, our present study showed the transcription of the defense-related genes in peach fruit was not induced by BABA treatment alone, only in fruit that were both treated with BABA and inoculated with *R. stolonifera* was a significant increase in these genes expression observed. Therefore, our results indicated that BABA induced disease resistance against *R. stolonifer* via priming.

Non-expressor of pathogenesis-related protein1 is a key regulator in plant immune system which activates the expression of PR genes (Kinkema et al., 2000). Luna et al. (2014) found that BABA primed disease resistance against the pathogens *Hyaloperonospora arabidopsidis* and *Pseudomonas syringae* pv. *tomato* DC3000 in *Arabidopsis* plants for up to 4 weeks after the treatment. This long-lasting priming was controlled by NPR1 and associated with priming of SA-inducible genes. In this study, BABA primed for augmented expression of defense-related genes including *NPR1-like* and enhanced disease resistance against *R. stolonifera* in peach fruit. However, the role of NPR1 on regulating the expression of PR genes in BABA-induced priming defense in harvested fruits needs further investigation.

## Conclusion

Our study indicated that BABA treatment primed induction of the resistance response to control *Rhizopus* rot development in post-harvest peaches by enhancing the expression of defense-related genes. BABA also induced activities of enzymes involved in lignin biosynthesis and energy metabolism pathways and thereby maintaining the strength of the cell wall and energy status in harvested peaches, which contributes to increase the disease resistance against *Rhizopus* rot.

## Author Contributions

JW and YZ conceived and designed the experiments. JW, LW, and XW performed gene expression and enzyme activity assays. JW and SC carried out ATP, ADP, AMP, lignin content, and analyzed the data. PJ and YZ contributed to reagents, materials, and analysis tools. JW, SC, PJ, LW, XW, and YZ participated in writing the manuscript. All the authors read and approved the final manuscript.

## Conflict of Interest Statement

The authors declare that the research was conducted in the absence of any commercial or financial relationships that could be construed as a potential conflict of interest.
